# Risk Assessment Related to Atmospheric Polycyclic Aromatic Hydrocarbons in Gas and Particle Phases near Industrial Sites

**DOI:** 10.1289/ehp.1002855

**Published:** 2011-04-08

**Authors:** Noelia Ramírez, Anna Cuadras, Enric Rovira, Rosa Maria Marcé, Francesc Borrull

**Affiliations:** 1 Department of Analytical Chemistry and Organic Chemistry, Universitat Rovira i Virgili, Tarragona, Spain; 2 Observatory of Health and Environment of Tarragona, Agència de Protecció de la Salut, Departament de Salut, Generalitat de Catalunya, Tarragona, Spain

**Keywords:** air quality, gas phase, particulate phase, polycyclic aromatic hydrocarbons, risk assessment

## Abstract

Background: Inhalation is one of the main means of human exposure to polycyclic aromatic hydrocarbons (PAHs) because of their ubiquitous presence in the atmosphere. However, most studies have considered only PAHs found in the particle phase and have omitted the contribution of the gas-phase PAHs to the risk.

Objective: We estimated the lifetime lung cancer risk from PAH exposure by inhalation in people living next to the largest chemical site in Southern Europe and the Mediterranean area.

Methods: We determined 18 PAHs in the atmospheric gas and particle phase. We monitored the PAHs for 1 year in three locations near the chemical site in different seasons. We used toxic equivalence factors to calculate benzo[*a*]pyrene (BaP) equivalents (BaP-eq) for individual PAHs and applied the World Health Organization unit risk (UR) for BaP (UR = 8.7 × 10^–5^) to estimate lifetime cancer risks due to PAH exposures.

Results: We observed some spatial and seasonal variability in PAH concentrations. The contribution of gas-phase PAHs to the total BaP-eq value was between 34% and 86%. The total estimated average lifetime lung cancer risk due to PAH exposure in the study area was 1.2 × 10^–4^.

Conclusions: The estimated risk was higher than values recommended by the World Health Organization and U.S. Environmental Protection Agency but lower than the threshold value of 10^–3^ that is considered an indication of definite risk according to similar risk studies. The results also showed that risk may be underestimated if the contributions of gas-phase PAHs are not considered.

Polycyclic aromatic hydrocarbons (PAHs) are a large group of organic contaminants, characterized by the presence of at least two fused aromatic rings. These compounds result from pyrolytic processes and the incomplete combustion of organic matter at high temperatures, and anthropogenic activities are the main source of PAHs in the environment. Because of their adverse effects on human health, their persistence in environmental matrices, and their reactivity and ability to transform into more active species, PAHs have been classified as priority pollutants by both the U.S. Environmental Protection Agency (EPA 1994) and the European Environment Agency (1999).

PAH health effects have been widely studied, primarily because of their potential carcinogenic and mutagenic properties. Several toxicological studies in animals [World Health Organization–International Programme on Chemical Safety (WHO–IPCS) 1998] and occupational studies in humans (Armstrong et al. 2004) demonstrate an excess risk of lung cancer associated with PAH inhalation. The potential influence of PAH exposure on the development of bladder and urinary system cancer also has been studied (Bosetti et al. 2007). In general, the carcinogenic properties of PAHs increase with the number of aromatic rings. Benzo[*a*]pyrene (BaP) has been the most extensively studied PAH and is the usual marker for carcinogenic levels of PAHs in environmental studies. However, uncertainties about the suitability of BaP as a cancer risk indicator have also been discussed (Boström et al. 2002). The International Agency for Research on Cancer (IARC) classified BaP as carcinogenic to humans (group 1); other PAHs, such as dibenzo[*a*,*h*]anthracene (DahA), as probably carcinogenic to humans (group 2A); and other PAHs, such as naphthalene (Nap), benzo[*a*]anthracene (BaA), chrysene (Chr), benzo[*b*]fluoranthene (BbF), benzo[*j*]fluoranthene (BjF), and indeno[1,2,3-*cd*]pyrene (Ind) as possibly carcinogenic to humans (group 2B). PAHs can also metabolize and become reactive electrophilic intermediates that can form DNA adducts, which may induce mutations and ultimately tumors (IARC 2010). Some PAHs, such as fluoranthene (FluT), which IARC classified as weak carcinogens (group 3), have mutagenic characteristics and may therefore play an important role in carcinogenesis (Boström et al. 2002).

Studies of other outcomes have suggested effects of PAHs on the development of arteriosclerosis (WHO 2000), reproductive outcomes such as intrauterine growth retardation (Dejmek et al. 2000; Šrám et al. 2005), and children’s neurological development (Perera et al. 2009). Furthermore, because PAHs are highly hydrophobic, they have an affinity for environmental matrices such as sediments, soils, and biota and can bioaccumulate in adipose tissues and become magnified through the food chain (WHO–IPCS 1998). Because of their lipophilic characteristics and limited biodegradation, PAHs are classified as persistent organic pollutants.

Ingestion is quantitatively the main route for PAH human exposure. However, inhalation is also a significant route because of the ubiquitous presence of these compounds in the atmosphere [Agency for Toxic Substances and Disease Registry (ATSDR) 1995; Li et al. 2010]. PAHs can be associated with the atmospheric gas phase and particulate phase (Ravindra et al. 2008; WHO–IPCS 1998). The most lipophilic compounds are mainly associated with the particle fraction, which can accumulate in the tracheobronchial epithelium, where they increase PAH concentrations even at low environmental PAH exposures (Boström et al. 2002; Gerde et al. 1993). In contrast, PAHs present in the gas phase or those that rapidly elute from particles upon inhalation can reach the alveolar epithelium and rapidly enter the circulatory system (Boström et al. 2002; Gerde et al. 1993).

Risk estimation for PAH exposures is complex for several reasons. There are few reported human epidemiological studies of individual PAHs, and individual PAHs are likely to induce cancer through different mechanisms. As mentioned above, BaP is the most studied PAH, and other PAHs have been ranked according to cancer potency relative to BaP using toxic equivalence factors (TEFs). The application of TEFs combined with the WHO quantitative risk assessment (QRA) methodology (WHO 2000) can be used to estimate the excess lifetime risk of lung cancer due to PAH exposures. This methodology has been used to establish guideline values for PAH exposures and inform environmental policies (Morello-Frosch et al. 2000).

The aim of the present study is to estimate the lifetime lung cancer risk of PAH exposure by inhalation in people living in urban and suburban areas near an important industrial chemical site in the Tarragona region (northeastern Spain). To that end, we selected 18 PAH compounds [most of them included in the U.S. EPA Priority List (ATSDR 2007)], in both the gas and particle phases. We monitored three locations near the two industrial complexes in the region from June 2008 to June 2009. We evaluated the distribution of PAHs in the gas and particle phase, their seasonal and spatial distributions, and the contribution of individual PAHs to estimated risks.

Few studies related to industrial atmospheres consider the contribution of both the atmospheric gas phase and the particle phase to risk (Liu et al. 2010). To our knowledge, this is the first study to estimate lifetime lung cancer risk due to inhalation of PAHs in both the gas and particle phases among people living near a petrochemical and chemical industrial site with these characteristics.

## Materials and Methods

*Study area.* The largest chemical site in Southern Europe and the Mediterranean area is located in the Tarragona region (European Chemical Regions Network 2010). Most of the chemical industries are located in two areas known as the North and the South Industrial Complexes. The North Industrial Complex has an area of 470 ha and includes an oil refinery and other chemical industries. Among the products manufactured in the North Complex are benzene, ethylene, fuel oil, gasoline, kerosene, propylene, propylene oxide, polypropylene, and styrene. The South Industrial Complex occupies an area of 717 ha and includes several chemical and petrochemical plants. Among the products manufactured in the South Industrial Complex are acrylonitrile-butadiene-styrene (ABS), butane, chlorine, ethylene oxide, polyethylene, kerosene, halogenated organic compounds, polyols, polypropylene, polystyrene, polyethylene, polyvinyl chloride, propane, and vinyl acetate. The total production of the two complexes is about 21 million tons per year (refining, 8.3 million tons; chemical products, 12.7 million tons [Associació Empresarial Química de Tarragona (AEQT) 2009]).

**Figure 1:**
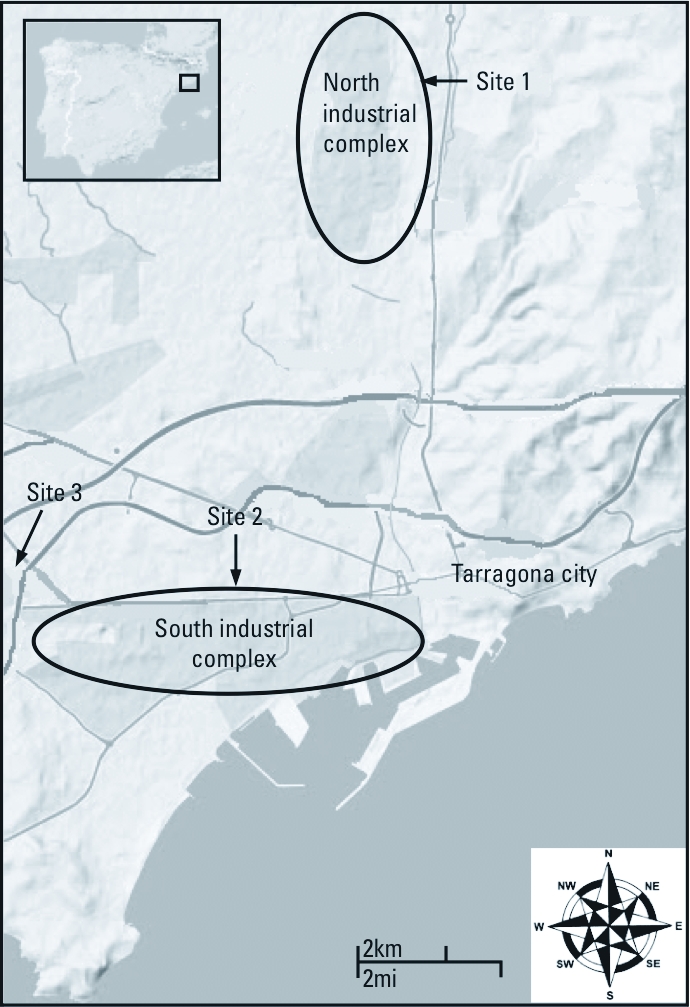
Map of the Tarragona region showing the location of the main city (Tarragona), the North and South Industrial Complexes, and the three sampling points: site 1 (Perafort-Puigdelfí) near the North Industrial Complex and site 2 (Tarragona-Bonavista) and site 3 (Vila-seca) near the South Industrial Complex.

Figure 1 shows the map of the study area. We selected three sampling sites on the basis of their proximity to the industrial complexes and the prevailing wind. Site 1 is located in Perafort-Puigdelfí, a semirural area < 0.5 km east of the North Industrial Complex. Site 2, Tarragona-Bonavista, is in a suburban area of the main city of Tarragona < 1 km north of the South Industrial Complex and < 0.5 km from a road with a moderate traffic density [14,041 vehicles per day (Spanish Ministry of Public Works, personal communication)]. Finally, site 3 is also located in Vila-seca, a suburban area < 1 km from the west end of the South Industrial Complex and < 1 km from a road with a moderate traffic density (20,049 vehicles per day (Spanish Ministry of Public Works, personal communication). Wind speeds are light (annual average < 3 m/sec), and the prevailing wind directions are north-northwesterly in cold seasons and south-southeasterly in warm seasons.

*Sample collection.* We collected samples using a TE-1000 polyurethane foam (PUF) high-volume air sampler (Tisch Environmental, Inc., Village of Cleves, OH, USA), which simultaneously collects total suspended particles and airborne pollutant vapors, meeting the specifications of U.S. EPA method TO-13 (U.S. EPA 1999). We took samples for 24 hr at a flow rate of approximately 0.2 m^3^/min on quartz microfiber filters (QFFs; 10.16 cm diameter, grade QM-A; Whatman, Maidstone, UK) for trapping particulate matter and PUF cartridges (63 mm diameter, 76 mm long; Tisch) for gas-phase pollutants. Before the sampling step, we conditioned the QFFs at 400°C for at least 6 hr, and the PUFs using dichloromethane (DCM) Soxhlet extraction for 24 hr and then dried them in a vacuum desiccator. After the conditioning step, we individually wrapped the QFFs and PUFs in aluminum foil, protected them in a sealable plastic bag, and kept them at –18°C before and after the sampling, until analysis. We analyzed the samples within 2 weeks of collection.

We conducted the sampling campaign over 1 year between June 2008 and June 2009. During this period, we took a total of 153 samples of matched air and particulate matter in three weekly samples over approximately 4 months in each location (52 samples at site 1, 48 samples at site 2, and 53 samples at site3). We collected samples during two phases in each location, including 2 months during a high-temperature period and 2 months during a low-temperature period, to determine whether temperature may influence PAH values in the atmosphere.

*Chemical standards and reagents.* We measured 18 target PAHs and used five deuterated PAHs as internal standards. Standards for Nap, acenaphthylene (AcPy), acenaphthene (AcP), fluorene (Flu), phenanthrene (PA), anthracene (Ant), FluT, pyrene (Pyr), BaA, Chr, BbF, benzo[*k*]fluoranthene (BkF), BaP, and DahA were supplied by Sigma-Aldrich (St. Louis, MO, USA). Standards for BjF, benzo[*e*]pyrene (BeP), Ind, benzo[*g*,*h*,*i*]perylene (BghiP), and the deuterated PAHs (d_8_-Nap, d_10_-AcP, d_10_-PA, d_12_-Chr, and d_12_-perylene) were supplied by Supelco (Bellefonte, PA, USA). Dimethylformamide (DMF) was provided by Merck (Darmstadt, Germany). The purity of all standards was > 96%.

DCM with purity > 99.9% (SDS, Peypin, France) was used to extract the analytes and prepare the standard dilutions. Helium and nitrogen gas (99.999% purity) were used for the chromatographic analysis and extractions, respectively (Carburos Metálicos, Barcelona, Spain), and Hyflo Super Cel diatomaceous earth (Sigma-Aldrich) was used to fill the QFF extraction cells of the pressurized liquid extraction (PLE) equipment. Diatomaceous earth was conditioned at 400°C for 6 hr and then kept in a desiccator.

*Sample extraction.* We extracted PAHs by PLE using Accelerate Solvent Extraction ASE 200 equipment (DIONEX, Sunnyvale, CA, USA) and DCM as solvent. For the pressurized solvent extraction of the PUFs, we cut them in two pieces using acetone-rinsed scissors and then placed them in 33-mL extraction cells. Extraction of the PUFs consisted of one cycle that began with a preheating step of 5 min and a static time of 5 min with a temperature of 100°C and a pressure of 1,500 psi. Flush volume was 50%, and purge time was 120 sec. We cut the QFFs into small pieces, mixed them with diatomaceous earth, and placed them in an 11-mL cell. Extraction of QFFs was performed in two cycles under the same conditions as the PUF extraction. The PLE extraction conditions of PUFs and QFFs were optimized in a previous study (Ras et al. 2009a).

We placed the extract from PLE in a 100-mL round-bottom flask and added 400 μL DMF to prevent the evaporation of the most volatile PAHs (Lintelmann et al. 2005). We reduced the extract to the DMF volume (400 μL) on a rotary evaporator and then transferred it to a 1-mL volumetric flask by adding DCM. We then added 20 μL of 50 mg/L deuterated PAH solution (to obtain an internal standard concentration of 1 mg/L), and the volumetric flask was filled to the mark with DCM. Recoveries of the extraction were > 90% for all target PAHs [see Supplemental Material, Table 1 (doi:10.1289/ehp.0902855)].

**Table 1:** Minimum, maximal, and average PAH concentrations (ng/m^3^) found in the three locations over the two seasonal sampling periods.

Site 1											Site 2											Site 3										
Summer					Winter					Summer					Winter–spring					Spring					Autumn				
Min	Max	Avg	Min	Max	Avg	Min	Max	Avg	Min	Max	Avg	Min	Max	Avg	Min	Max	Avg
Gas phase																																				
Nap		ND		3.53		1.37		0.24		20.4		3.92		ND		0.86		0.36		0.48		1.68		0.92		0.24		0.68		0.37		0.53		13.4		2.97
AcPy		ND		2.21		0.51		0.31		11.6		3.18		ND		0.60		0.23		0.33		1.29		0.74		ND		0.41		0.28		0.14		15.5		2.89
AcP		ND		1.88		0.33		0.15		5.77		1.26		ND		0.47		0.22		0.38		0.93		0.63		0.16		0.98		0.43		0.25		8.26		2.31
Flu		0.39		1.62		0.69		0.42		14.4		5.52		ND		0.84		0.41		0.70		2.10		1.42		0.53		2.66		1.35		0.24		33.0		6.95
PA		0.80		2.46		1.43		1.26		91.1		28.2		0.54		2.23		1.13		1.69		8.57		4.15		1.29		25.3		12.1		0.31		197		35.2
Ant		ND		1.58		0.64		0.19		9.00		2.66		ND		2.27		0.35		0.29		0.70		0.46		0.11		1.07		0.46		ND		17.1		2.43
FluT		0.29		0.94		0.56		0.32		20.4		6.01		0.43		1.18		0.64		0.40		1.74		0.82		0.38		4.39		2.03		0.24		43.3		5.84
Pyr		ND		0.83		0.38		0.24		24.1		6.31		ND		0.21		0.05		0.38		1.13		0.76		ND		2.17		0.65		0.23		32.9		4.93
BaA		ND		1.11		0.30		ND		1.38		0.58		ND		0.35		0.08		ND		0.46		0.20		ND		0.30		0.27		ND		6.37		0.77
Chr		ND		1.26		0.25		0.07		7.43		1.30		ND		0.20		0.02		ND		0.43		0.23		ND		0.45		0.35		ND		2.94		0.46
BbF		ND		1.56		0.35		ND		0.13		ND		ND		0.78		0.09		ND		ND		ND		ND		ND		ND		ND		9.29		0.63
BjF/BkF		ND		1.79		0.78		ND		0.66		0.14		ND		0.95		0.64		ND		ND		ND		ND		0.04		0.04		ND		1.11		0.21
BeP		ND		0.17		0.01		ND		0.36		0.04		ND		0.33		0.15		ND		ND		ND		ND		0.13		0.09		ND		2.11		0.27
BaP		ND		1.11		0.29		ND		0.37		0.03		ND		0.35		0.17		ND		ND		ND		ND		ND		ND		ND		3.06		0.26
Ind		ND		1.15		0.32		ND		0.59		0.09		ND		0.45		0.25		ND		ND		ND		ND		0.44		0.32		ND		2.78		0.33
DahA		ND		5.34		0.50		ND		0.89		0.17		ND		0.77		0.51		ND		0.57		0.02		ND		0.71		0.60		ND		3.82		0.46
BghiP		ND		2.19		0.29		ND		0.74		0.09		ND		0.35		0.23		ND		ND		ND		ND		0.51		0.32		ND		3.00		0.32
Total PAH		2.13		28.5		8.99		3.60		167		59.5		2.72		8.48		5.52		5.26		18.2		10.4		4.63		34.6		17.8		2.14		361		67.2
Particle phase																																				
Nap		ND		3.28		0.24		ND		0.11		0.04		ND		0.72		0.16		ND		0.72		0.21		ND		0.28		0.07		ND		0.33		0.14
AcPy		ND		2.52		0.10		ND		0.10		0.01		ND		0.54		0.12		ND		0.24		0.04		ND		ND		ND		ND		0.12		ND
AcP		ND		1.53		0.11		ND		0.17		0.04		ND		0.59		0.16		ND		0.31		0.14		ND		0.43		0.09		ND		0.42		0.05
Flu		ND		0.95		0.08		ND		0.31		0.08		ND		0.40		0.08		ND		0.36		0.05		ND		ND		ND		ND		0.13		0.01
PA		ND		1.11		0.26		ND		1.37		0.37		ND		0.32		0.15		ND		0.15		0.01		ND		0.68		0.04		ND		0.17		0.06
Ant		ND		1.45		0.26		ND		0.44		0.20		ND		0.36		0.12		ND		0.31		0.20		ND		0.27		0.04		ND		0.14		0.01
FluT		ND		0.88		0.17		ND		2.28		0.51		ND		0.50		0.31		ND		0.28		0.12		ND		0.41		0.07		ND		0.37		0.11
Pyr		ND		3.44		0.38		0.32		1.57		0.63		ND		0.17		0.05		ND		0.39		0.26		ND		0.21		0.05		ND		0.34		0.15
BaA		ND		2.67		0.37		0.12		3.44		0.99		ND		0.32		0.11		0.20		0.59		0.34		ND		0.30		0.19		ND		1.05		0.17
Chr		ND		3.44		0.39		0.11		1.88		0.75		ND		0.17		0.04		0.27		0.49		0.38		ND		0.45		0.09		ND		1.25		0.17
BbF		ND		2.67		0.44		ND		0.07		ND		ND		0.37		0.11		ND		ND		ND		ND		0.04		ND		ND		0.13		0.01
BjF/BkF		ND		2.39		1.05		ND		1.93		1.03		ND		1.57		0.75		ND		0.20		0.04		ND		ND		ND		ND		1.63		0.27
BeP		ND		0.12		0.06		0.14		0.38		0.23		ND		0.50		0.25		ND		ND		ND		ND		0.17		0.03		ND		0.17		0.06
BaP		ND		1.36		0.39		0.14		1.53		0.84		ND		0.45		0.24		ND		0.46		0.02		ND		ND		ND		ND		0.46		0.09
Ind		ND		2.25		0.48		ND		0.96		0.60		ND		0.76		0.35		ND		0.45		0.10		ND		ND		ND		ND		0.19		0.02
DahA		ND		1.86		0.42		ND		0.87		0.26		ND		0.82		0.54		ND		ND		ND		ND		ND		ND		ND		ND		ND
BghiP		ND		1.53		0.47		ND		1.20		0.65		ND		0.55		0.29		ND		0.37		0.10		ND		ND		ND		ND		0.28		0.07
Total PAH		0.61		33.3		5.61		1.08		14.4		7.00		0.46		7.71		3.82		1.16		3.72		2.01		ND		1.65		0.67		ND		5.94		1.39
Sampling period		02/06/08–30/07/08						01/12/08–04/02/09						04/08/08–25/09/08						11/02/09–08/04/09						20/04/09–17/06/09						29/09/08–26/11/08				
No. samples		26						26						22						26						26						27				
Average temperature		23.2 ± 2.5						9.0 ± 2.5						25.0 ± 2.7						12.0 ± 1.5						20.5 ± 2.8						15.5 ± 4.3				
Percent PAHs Air/PM		62.0/38.0						89.5/10.5						59.1/40.9						83.8/16.2						96.4/3.6						98.0/2.0				
Abbreviations: Avg, average; Max, maximum; Min, minimum; ND, not detected (values under the limit of detection); PM, particulate matter.																																				

*Gas chromatography–mass spectrometry analysis.* We analyzed extracts in a 6890N gas chromatograph coupled with a 5973 inert mass spectrometer (Agilent Technologies, Palo Alto, CA, USA), using a capillary column Zebron ZB-5 (5% phenyl, 95% dimethyl polysiloxane, 30 m × 0.25 mm i.d. × 0.25 μm; Phenomenex, Le Pecq Cedex, France). The gas chromatography–mass spectrometry (GC-MS) analysis was performed by injecting 1 μL extract at 270°C, in splitless mode, at a helium constant flow of 1 mL/min. The oven temperature program started at 100°C, held for 4 min, was raised to 290°C at a rate of 6°C/min, and held there for 10 min.

The GC-MS interface was set at 280°C. The mass spectrometer acquired data in scan mode with an *m*/*z* interval from 35 to 280, operating at electron impact energy of 70 eV. Qualitative identification of target PAHs was based on the match of the retention times and the ion ratios of the target quantifier and qualifier ions. We quantified the isomers BjF and BbF together because they coelute and exhibit similar mass spectra.

We used the internal standard calibration method for GC-MS quantification. The standards, diluted in DCM, ranged between 0.01 and 50 mg/L with an internal standard constant concentration of 1 mg/L. We prepared all the standards on the day of use and kept them refrigerated. For an average air preconcentration of 300 m^3^, limits of detection (LODs) ranged from 0.002 ng/m^3^ to 0.033 ng/m^3^, and limits of quantification (LOQs) from 0.033 ng/m^3^ to 0.167 ng/m^3^. Repeatability and reproducibility were below the 10% relative standard deviation (RSD) (*n* = 5) for all target compounds. For further information about the validation parameters of the analytical method, see Supplemental Material, Table 1 (doi:10.1289/ehp.0901233).

*Quality assurance.* Because of the ubiquitous presence of PAHs in the atmosphere, precautions to avoid sample contamination were taken throughout the entire process. In addition to the precautions mentioned previously, we followed U.S. EPA method TO-13 (U.S. EPA 1999) guidelines. Furthermore, components of the extraction cells and collecting vials were ultrasound-cleaned with isopropanol followed by DCM and dried inside a fume cupboard. All glassware, including GC syringes, was rinsed three times with isopropanol and three times with DCM before use. Field blanks, process blanks, and solvent blanks were also performed according to section 14 of method TO-13. Taking these precautions into account, no blank showed any detectable signals for the target PAHs.

*Toxic equivalency factors: BaP equivalency.* For risk assessment calculations, we ranked the 18 target PAHs according to their cancer potency relative to BaP. Most of the TEFs used in this study were proposed by Larsen and Larsen (1998) based on current knowledge of PAH carcinogen effects. However, for volatile PAHs such as Nap, AcPy, and AcP, we applied TEFs proposed by Nisbet and Lagoy (1992).

We calculated BaP equivalents (BaP-eq) by multiplying each individual PAH concentration with its corresponding TEF and determined the concentration of total PAH expressed as BaP-eq. Following U.S. EPA criteria (U.S. EPA 2000) for risk assessment calculations, we substituted half the LOD for measured values < LODs and half the LOQ value for measured values < LOQs.

*Lifetime lung cancer risk of PAHs in the atmosphere defined by the WHO unit risk.* The estimated lifetime lung cancer risk from PAHs in the atmosphere based on the WHO unit risk (UR) (WHO 2000) is 8.7 cases per 100,000 people with chronic inhalational exposure to 1 ng/m^3^ BaP (UR = 8.7 × 10^–5^) over a lifetime of 70 years.

The risk of developing lung cancer can thus be calculated as

Lifetime lung cancer risk =   BaP-eq (ng/m^3^) × UR. [1]

We estimated the corresponding annual number of lung cancer cases in the population around the chemical site that could be attributed to PAH as follows: population exposed (number of inhabitants) × lifetime lung cancer risk ÷ 70 (years of exposure). We defined the exposed population for this study (170,263 inhabitants) based on the proximity to the industrial complexes and the prevailing winds.

## Results and Discussion

*PAH gas and particle phase values.* PAHs in air can be present either in the gas phase or in the particulate phase. The phase distribution of the PAHs depends on the vapor pressure of the compound, the atmospheric temperature, the PAH concentration, the affinity of the PAH for the atmospheric suspended particles (determined by the partitioning coefficients *K*_oc_), and the nature and concentrations of the particles (ATSDR 1995; Baek et al. 1991). Table 1 summarizes the PAH concentrations we determined. In general, PA showed the highest average concentration in the gas phase. Nap, Flu, FluT, and Pyr also were common gas-phase PAHs, whereas BaA, BjF, BkF, and Chr formed most of the particle phase. Temperature appeared to have a considerable influence on PAH concentrations because the highest levels of PAHs occurred in winter, with Nap, AcPy, AcP, Flu, PA, Ant, FluT, BaA, and Chr increasing at lower temperatures. Furthermore, in samples collected during the low-temperature periods, the heaviest PAHs (BbF, BjF, BkF, BeP, BaP, Ind, DahA, BghiP) were present mainly in the particulate phase, whereas a more equitable distribution of these compounds in both phases was observed in samples collected during the high-temperature periods. As an example, Supplemental Material, Tables 2 and 3 (doi:10.1289/ehp.0902855), presents the daily PAH concentrations obtained in site 1 over high- and low-temperature periods. For instance, in samples collected at site 1 during the winter, BaP concentrations in gas-phase samples ranged from values under the LOD (ND) of each sample to 0.37 ng/m^3^ and in particle-phase samples ranged from 0.14 to 1.53 ng/m^3^, whereas in summer concentrations ranged from ND and 1.11 ng/m^3^ in the gas phase and from ND and 1.36 ng/m^3^ in the particle phase. One possible explanation is that when the temperature is high, some particle-bound PAHs may evaporate and be trapped in the PUFs during sampling (U.S. EPA 1999).

**Table 2:** Average BaP-eq (ng/m^3^) for gas and particle phases in the three sampling sites, during two seasonal sampling periods.

Site 1 (gas/particle phase)			Site 2 (gas/particle phase)			Site 3 (gas/particle phase)		
PAH	TEF*a*	Summer	Winter	Summer	Winter–spring	Spring	Autumn
Nap		0.001		1.4 × 10^–3^ / 2.5 × 10^–4b^		4.2 × 10^–3^ / 5.0 × 10^–5^		3.7 × 10^–4^ / 1.6 × 10^–4^		9.2 × 10^–4^ / 2.1 × 10^–4^		3.7 × 10^–4^ / 7.0 × 10^–5^		3.0 × 10^–3^ / 1.3 × 10^–4^
AcPy		0.001		5.2 × 10^–4^ / 1.9 × 10^–4b^		3.4 × 10^–3^ / 3.0 × 10^–5^		2.3 × 10^–4^ / 1.3 × 10^–4^		7.4 × 10^–4^ / 5.0 × 10^–5^		1.2 × 10^–4^ / 2.0 × 10^–5b^		3.0 × 10^–3^ / 2.5 × 10^–5b^
AcP		0.001		3.1 × 10^–4^ / 1.2 × 10^–4b^		1.3 × 10^–3^ / 4.0 × 10^–5^		2.1 × 10^–4^ / 1.7 × 10^–4^		6.3 × 10^–4^ / 1.5 × 10^–4^		4.2 × 10^–4^ / 1.0 × 10^–4^		2.2 × 10^–3^ / 5.0 × 10^–5^
Flu		0.0005		3.5 × 10^–4^ / 5.0 × 10^–5^		3.0 × 10^–3^ / 3.5 × 10^–5^		2.1 × 10^–4^ / 4.5 × 10^–5^		7.1 × 10^–4^ / 3.0 × 10^–5^		6.8 × 10^–4^ / 8.0 × 10^–6b^		3.6 × 10^–3^ / 2.5 × 10^–5b^
PA		0.0005		7.1 × 10^–4^ / 1.3 × 10^–4^		1.5 × 10^–2^ / 1.9 × 10^–4^		5.7 × 10^–4^ / 7.5 × 10^–5^		2.1 × 10^–3^ / 2.0 × 10^–5^		6.2 × 10^–3^ / 3.0 × 10^–5b^		1.9 × 10^–2^ / 3.5 × 10^–5^
Ant		0.0005		3.4 × 10^–4^ / 1.4 × 10^–4^		1.4 × 10^–3^ / 9.5 × 10^–5^		1.8 × 10^–4^ / 6.5 × 10^–5^		2.3 × 10^–4^ / 1.0 × 10^–4^		2.3 × 10^–4^ / 3.0 × 10^–5^		1.3 × 10^–3^ / 2.5 × 10^–5b^
FluT		0.05		2.8 × 10^–2^ / 8.0 × 10^–3^		0.32 / 2.5 × 10^–2^		3.2 × 10^–2^ / 1.5 × 10^–2^		4.1 × 10^–2^ / 6.0 × 10^–3^		0.11 / 3.5 × 10^–3^		0.31 / 5.5 × 10^–3^
Pyr		0.001		3.8 × 10^–4^ / 3.8 × 10^–4^		6.8 × 10^–3^ / 6.2 × 10^–4^		5.0 × 10^–5^ / 5.0 × 10^–5^		7.6 × 10^–4^ / 2.0 × 10^–4^		6.4 × 10^–4^ / 6.0 × 10^–5^		5.3 × 10^–3^ / 1.5 × 10^–4^
BaA		0.005		1.5 × 10^–3^ / 1.9 × 10^–3^		3.1 × 10^–3^ / 4.6 × 10^–3^		4.0 × 10^–4^ / 5.5 × 10^–4^		1.0 × 10^–3^ / 1.7 × 10^–3^		2.7 × 10^–4^ / 1.0 × 10^–4^		4.3 × 10^–3^ / 8.5 × 10^–4^
Chr		0.03		7.5 × 10^–3^ / 1.2 × 10^–2^		4.2 × 10^–2^ / 2.3 × 10^–2^		6.0 × 10^–4^ / 1.2 × 10^–3^		6.9 × 10^–3^ / 1.1 × 10^–2^		2.1 × 10^–3^ / 3.0 × 10^–3^		1.5 × 10^–2^ / 5.4 × 10^–3^
BbF		0.1		3.5 × 10^–2^ / 4.6 × 10^–2^		2.0 × 10^–3b^ / 2.0 × 10^–3^		9.0 × 10^–3^ / 1.2 × 10^–2^		1.0 × 10^–3b^ / 1.0 × 10^–3b^		1.0 × 10^–3b^ / 1.0 × 10^–3b^		7.1 × 10^–2^ / 2.0 × 10^–3^
BjF/BkF		0.05		3.9 × 10^–2^ / 4.9 × 10^–2^		7.5 × 10^–3^ / 5.2 × 10^–2^		3.2 × 10^–2^ / 3.7 × 10^–2^		5.0 × 10^–5b^ / 2.0 × 10^–3^		1.5 × 10^–4b^ / 1.5 × 10^–4b^		1.1 × 10^–2^ / 1.5 × 10^–2^
BeP		0.002		2.0 × 10^–5^ / 1.8 × 10^–4b^		8.0 × 10^–5b^ / 4.8 × 10^–4^		3.6 × 10^–4^ / 5.8 × 10^–4^		2.0 × 10^–6b^ / 2.0 × 10^–5b^		1.8 × 10^–5b^ / 6.6 × 10^–5^		5.8 × 10^–4^ / 1.4 × 10^–4^
BaP		1		0.30 / 0.40		5.0 × 10^–2^ / 0.85		0.15 / 0.21		2.0 × 10^–2b^ / 6.0 × 10^–2b^		2.7 × 10^–2b^ / 3.8 × 10^–3b^		0.28 / 0.14
Ind		0.1		3.2 × 10^–2^ / 4.7 × 10^–3^		1.0 × 10^–2^ / 6.1 × 10^–2^		2.5 × 10^–2^ / 3.5 × 10^–2^		2.0 × 10^–3b^ / 1.3 × 10^–2^		6.4 × 10^–3^ / 1.6 × 10^–3b^		3.9 × 10^–2^ / 8.0 × 10^–3^
DahA		1.1		0.56 / 0.48		0.21 / 0.27		0.56 / 0.59		4.4 × 10^–2b^ / 4.4 × 10^–2b^		0.12 / 1.8 × 10^–2b^		0.58 / 4.4 × 10^–2b^
BghiP		0.02		6.0 × 10^–3^ / 9.4 × 10^–3^		2.0 × 10^–3^ / 1.3 × 10^–2^		4.6 × 10^–3^ / 6.0 × 10^–3^		4.0 × 10^–4b^ / 2.6 × 10^–3^		1.3 × 10^–3^ / 3.2 × 10^–4b^		7.6 × 10^–3^ / 2.4 × 10^–3^
Total PAHs (gas/PM)				1.014 / 1.059		0.685 / 1.307		0.817 / 0. 913		0.122 / 0.143		0.272 / 0.067		1.363 / 0.223
Total PAHs (gas + PM)				2.073		1.992		1.730		0.265		0.339		1.586
PM, particulate matter. **a**TEFs for Nap, AcPy, and AcP are from Nisbet and Lagoy (1992); all others are from Larsen and Larsen (1998). **b**Less than 15% of the samples quantified.														

Although low temperatures facilitate the condensation of volatile and semivolatile PAHs onto preexisting particles, we found that PAH concentrations in the gas phase increased more than those in the particle phase when temperatures decreased (Table 1). This trend has also been reported in previous studies (Cincinelli et al. 2007; Ravindra et al. 2006). One possible explanation may be that photochemical reactions with OH (hydroxyl) and NO_3_ (nitrate) radicals increase with higher temperatures. However, other factors, such as routine plant maintenance and seasonal holidays, may also influence seasonal variation in PAH distributions.

In general, the highest total PAH values were associated with the gas phase in both sampling periods, especially in winter (Table 1). This trend has been detected in previous studies (Ravindra et al. 2006). These concentrations in the gas phase may be even higher because the trapping efficiency of PUFs for the two- to three-ring PAHs is about 35% (U.S. EPA 1999).

European legislation only regulates some heavy PAHs (BaA, Chr, BbF, BjF, BkF, BaP, BeP, DahA) in the particulate phase (European Economic Community 2005). Based on results we obtained in this study, the current legislation appears to underestimate the main contribution of PAHs to the atmosphere.

*Spatial distribution of PAHs.* The volatile PAHs (Nap, AcPy, AcP, Flu, PA, Ant) were most abundant at all three sampling sites, especially in the gas phase, and they made a large contribution to the total PAH values (between 49% and 84% of the gas phase and between 11% and 36% of the particle phase; Table 1). Site 1 presented the highest average concentrations of PAHs in the particle phase, with an average total PAH concentration of 5.61 ng/m^3^ in summer and 7.00 ng/m^3^ in winter. Of particular interest are the high concentrations of some of the heavy PAHs in the gas phase, such as the maximum concentrations of DahA or BghiP (5.34 ng/m^3^ and 2.19 ng/m^3^, respectively). Because site 1 is a rural area, the PAH values primarily may reflect activities in the North Industrial Complex, particularly refining, which can be an important source of particulate phase bounded PAHs.

Site 3 presented the highest average concentrations for the gas phase, with average total PAH values of 17.8 ng/m^3^ in the high-temperature season and 67.2 ng/m^3^ in the low-temperature season, whereas site 2 presented the lowest average values in both phases (Table 1). These sites are suburban areas near the South Industrial Complex and roads with moderate traffic intensity. The high maximal PAH values found in the gas phase in site 3 could be reflect both traffic intensity and industrial activities in the South Industrial Complex.

The average total PAH levels of the industrial sites of this study were comparable with those measured in industrial sites and in suburban areas in Europe (Cincinelli et al. 2007; Ravindra et al. 2006), much higher than those previously reported for some urban areas (Ras et al. 2009b), and very much higher than those reported in high mountain regions of Europe (Fernández et al. 2002). The PAH levels of the sampling sites therefore seem to be related to the activities of the petrochemical industry in the vicinity.

*BaP-eq values of particle- and gas-phase PAHs.* Table 2 shows the average BaP-eq values in the gas phase and in the particle phase, for the 18 target PAHs and the total PAHs, by site and sampling period, and the TEFs applied for the 18 PAHs. We also indicate the individual PAH compounds that we quantified in < 15% of the samples.

The contribution of the gas phase to the total BaP-eq values in this study was substantial, with values ranging between 34% and 86%. This high contribution was due to the presence of some heavy PAHs in the gas phase with high TEFs, and the high concentrations of the most volatile PAHs in the gas phase.

It is worth noting that BaP-eq values for the particle phase were higher than those for the gas phase in winter in site 1 but comparable between the particle and gas phases in summer at this site, and comparable between the two phases during both seasons at site 2 (Table 2). For site 3, the major contribution to total BaP-eq came from the gas phase because of the low concentrations of heavy PAHs, such as BaP and DahA, in particle-phase samples from this site. Site 1 showed the highest total BaP-eq for the combined gas and particle phases in summer (2.073 ng/m^3^) and in winter (1.992 ng/m^3^).

**Figure 2:**
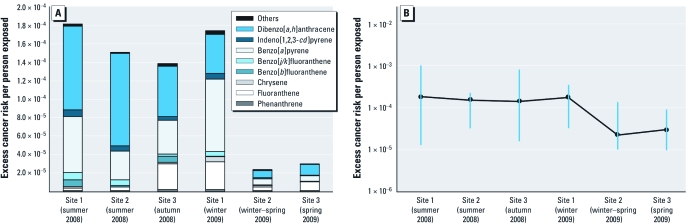
(*A*) Average estimated lifetime lung cancer risk expressed as excess cancer risk per person exposed, by sampling location, by period and PAH. (*B*) Minimum, maximum, and average estimated excess lifetime lung cancer risk per person exposed, in the three sampling sites by period. The vertical lines indicate the ranges of risk obtained with the daily concentrations, and the points mark the average risks.

*Lifetime lung cancer risk of 18 PAHs in the atmosphere.* Figure 2A shows the average lifetime lung cancer risk estimated for each PAH, by sampling site and sampling period. Site 1 showed the highest estimated risk with values of 1.8 × 10^–4^ (1.8 additional cases per 10,000 people exposed) for the summer period and 1.7 × 10^–4^ for the winter period. In the 2008 sampling periods, site 2 and site 3 had estimated lifetime lung cancer risks of 1.5 × 10^–4^ and 1.4 × 10^–4^, corresponding to a summer and autumn season, respectively, that are consistent with estimates for site 1. However, in the 2009 sampling periods the estimated risks for site 2 and site 3 were 2.2 × 10^–5^ (winter–spring) and 2.9 × 10^–5^ (spring), respectively, which were much lower than those estimated for 2008. These differences could reflect external factors, such as the international economic crisis that affected many industries located at the chemical site and probably caused reductions in production or shutdowns of some chemical plants (AEQT 2009), thus changing emissions.

Concerning the individual toxicity of the target PAHs, the compounds that contributed most to the total estimated risk were BaP (19–45%), DahA (24–67%), and FluT (2–32%; combined contribution, between 81% and 92%). The contribution of FluT that we found mainly in the gas phase and that had high values in site 1 in winter and in site 3 in autumn was also noteworthy. Although FluT is classified as a weak carcinogen, it is mutagenic and therefore has a high TEF (0.05). BbF, BjF, BkF, Chr, and Ind also contributed to the overall risk with maximum individual contributions between 4% and 7%. Although PA had the highest average concentration of the individual PAHs at the three sampling sites, it made a small contribution to the overall risk because of its low TEF.

Figure 2B shows the average, maximum, and minimum excess lifetime lung cancer risks per person exposed by period and sampling site. We estimated an average lifetime lung cancer risk of 1.2 × 10^–4^ (1.2 additional cases per 10,000 people exposed) for the study area as a whole. This represents the estimated risk above the baseline lifetime lung cancer risk in the absence of atmospheric PAH exposure. Taking into account the calculated average lifetime risk and assuming a homogeneous exposure of the inhabitants (170,263) around the chemical site, 0.3 annual cases of lung cancer can be attributed to this PAH exposure. Furthermore, in the absence of major indoor PAH sources, such as tobacco smoking, cooking fumes, and residential heating, outdoor PAHs can also substantially contribute to indoor PAH levels (Spengler et al. 2001).

According to the WHO “Air Quality Guidelines for Europe” (WHO 2000), each country has to determine its own acceptable risk levels. However, the risk estimate for the study area was higher than 10^–5^, the upper-bound excess lifetime cancer risk recommended by the WHO for carcinogens in drinking water (WHO 1993), and higher than the 10^–6^ risk that is the U.S. EPA guideline (Robson and Toscano 2007). We therefore conclude that the lung cancer risk due to these PAH exposures is not negligible and should be taken into account for health protection in the future.

Moreover, as shown in Figure 2B, the maximum risk we found was 1 × 10^–3^ in site 1. Even though this risk corresponds to one episode, this result is higher than the recommended values and would correspond to a definite risk according to criteria used in similar risk assessment studies (Sexton et al. 2007).

QRA is a powerful tool to derive a quantitative estimate of risk that can be used to inform environmental legislation. However, QRA has also areas of uncertainty that should be taken into account. There are two main uncertainties in the case of PAHs: the TEF values, which were established from toxicological animal studies, and the value of BaP UR, which was extrapolated from the results of epidemiological studies with exposure to high concentrations and can be biased in some cases, according to recent studies (Armstrong et al. 2004).

Finally, although the U.S. EPA has demonstrated that the reestimation of risk substituting the nondetected values with half the LOD and the nonquantified values with half the LOQ did not significantly affect risk assessment calculations by itself, the contribution of the compounds quantified in < 15% of the samples can be overestimated. In such cases, the use of statistical imputation methodologies for risk estimation has been suggested (U.S. EPA 2000).

## Conclusions

In this study, we determined 18 PAHs in 153 matched samples of atmospheric air and particulate matter from three sampling locations around a chemical site. PAH values were generally higher in low-temperature periods than in high-temperature periods, with higher individual PAH concentrations observed in the gas phase, especially for the most volatile PAHs.

Risk calculations showed that the contribution of the gas-phase PAHs to the total risk should be taken into account, as well as the contribution of volatile PAHs, such as FluT, and of the heaviest PAHs, such as BaP or DahA, especially during high-temperature periods.

The average estimated lifetime lung cancer risk for our study area was higher than the WHO and the U.S. EPA recommended values but lower than the threshold value of 10^–3^ considered a definite risk according to criteria used in similar risk assessment studies. Despite uncertainties associated with the sampling methodology and QRA calculations, our findings suggest that it would be prudent to take PAH concentrations in both gas and particle phases into account in future health-risk legislation. The real risk values may otherwise be underestimated.

## Supplemental Material

(164 KB) PDF

## References

[r1] AEQT (Associació Empresarial Química de Tarragona) (2009). Public Report 2009.

[r2] Armstrong B, Hutchinson E, Unwin J, Fletcher T. (2004). Lung cancer risk after exposure to polycyclic aromatic hydrocarbons: a review and meta-analysis.. Environ Health Perspect.

[r3] ATSDR (Agency for Toxic Substances and Disease Registry) (1995). Toxicological Profile for Polycyclic Aromatic Hydrocarbons (PAHS).. http://www.atsdr.cdc.gov/ToxProfiles/TP.asp?id=122&tid=25.

[r4] ATSDR (Agency for Toxic Substances and Disease Registry) (2007). CERCLA Priority List of Hazardous Substances.. http://www.atsdr.cdc.gov/cercla/07list.html.

[r5] Baek SO, Goldstone ME, Kirk PWW, Lester JN, Perry R (1991). Phase distribution and particle-size dependency of polycyclic aromatic-hydrocarbons in the urban atmosphere.. Chemosphere.

[r6] Bosetti C, Boffetta P, La Vecchia C. (2007). Occupational exposures to polycyclic aromatic hydrocarbons, and respiratory and urinary tract cancers: a quantitative review to 2005.. Ann Oncol.

[r7] Boström CE, Gerde P, Hanberg A, Jernström B, Johansson C, Kyrklund T (2002). Cancer risk assessment, indicators, and guidelines for polycyclic aromatic hydrocarbons in the ambient air.. Environ Health Perspect.

[r8] Cincinelli A, Del Bubba M, Martellini T, Gambaro A, Lepri L. (2007). Gas-particle concentration and distribution of n-alkanes and polycyclic aromatic hydrocarbons in the atmosphere of Prato (Italy).. Chemosphere.

[r9] Dejmek J, Solanský I, Benes I, Lenicek J, Šrám RJ (2000). The impact of polycyclic aromatic hydrocarbons and fine particles on pregnancy outcome.. Environ Health Perspect.

[r10] European Chemical Regions Network (2010). Catalonia.. http://www.ecrn.net/regions/cat.php.

[r11] European Economic Community 2005 Directive 2004/107/EC of the European Parliament and of the Council of 15 December 2004 Relating to Arsenic, Cadmium, Mercury, Nickel and Polycyclic Aromatic Hydrocarbons in Ambient Air. Off J Eur Commun 48:L23/3–L23/16.

[r12] European Environment Agency (1999). Criteria for EUROAIRNET: The EEA Air Quality Monitoring and Information Network. Technical Report No. 12.. http://www.eea.europa.eu/publications/TEC12.

[r13] Fernández P, Grimalt JO, Vilanova RM (2002). Atmospheric gas-particle partitioning of polycyclic aromatic hydrocarbons in high mountain regions of Europe.. Environ Sci Technol.

[r14] Gerde P, Muggenburg BA, Hoover MD, Henderson RF (1993). Disposition of polycyclic aromatic hydrocarbons in the respiratory tract of the beagle dog. 1. The alveolar region.. Toxicol Appl Pharmacol.

[r15] IARC (International Agency for Research on Cancer) (2010). Some non-heterocyclic polycyclic aromatic hydrocarbons and some related exposures.. Monogr Eval Carcinog Risk Hum.

[r16] Larsen JC, Larsen PB (1998). Chemical carcinogens.

[r17] Li Z, Mulholland JA, Romanoff LC, Pittman EN, Trinidad DA, Lewin MD (2010). Assessment of non-occupational exposure to polycyclic aromatic hydrocarbons through personal air sampling and urinary biomonitoring.. J Environ Monit.

[r18] Lintelmann J, Fischer K, Karg E, Schröppel A. (2005). Determination of selected polycyclic aromatic hydrocarbons and oxygenated polycyclic aromatic hydrocarbons in aerosol samples by high-performance liquid chromatography and liquid chromatography-tandem mass spectrometry.. Anal Bioanal Chem.

[r19] Liu HH, Yang HH, Chou CD, Lin MH, Chen HL (2010). Risk assessment of gaseous/particulate phase PAH exposure in foundry industry.. J Hazard Mater.

[r20] Morello-Frosch RA, Woodruff TJ, Axelrad DA, Caldwell JC (2000). Air toxics and health risks in California: the public health implications of outdoor concentrations.. Risk Anal.

[r21] Nisbet ICT, Lagoy PK (1992). Toxic equivalency factors (TEFs) for polycyclic aromatic-hydrocarbons (PAHs).. Regul Toxicol Pharmacol.

[r22] Perera FP, Li Z, Whyatt R, Hoepner L, Wang SA, Camann D (2009). Prenatal airborne polycyclic aromatic hydrocarbon exposure and child IQ at age 5 years.. Pediatrics.

[r23] Ras MR, Borrull F, Marcé RM (2009a). Pressurised liquid extraction of polycyclic aromatic hydrocarbons from gas and particulate phases of atmospheric samples.. J Sep Sci.

[r24] Ras MR, Marcé RM, Cuadras A, Mari M, Nadal M, Borrull F (2009b). Atmospheric levels of polycyclic aromatic hydrocarbons in gas and particulate phases from Tarragona Region (NE Spain).. Int J Environ Anal Chem.

[r25] Ravindra K, Bencs L, Wauters E, de Hoog J, Deutsch F, Roekens E (2006). Seasonal and site-specific variation in vapour and aerosol phase PAHs over Flanders (Belgium) and their relation with anthropogenic activities.. Atmos Environ.

[r26] Ravindra K, Sokhi R, Van Grieken R. (2008). Atmospheric polycyclic aromatic hydrocarbons: source attribution, emission factors and regulation.. Atmos Environ.

[r27] Robson MG Toscano WA, eds 2007 Risk Assessment for Environmental Health. San Francisco:Jossey-Bass.

[r28] Sexton K, Linder SH, Marko D, Bethel H, Lupo PJ (2007). Comparative assessment of air pollution-related health risks in Houston.. Environ Health Perspect.

[r29] Spengler JD McCarthy JF Samet JM 2001 Indoor Air Quality Handbook. New York:McGraw-Hill.

[r30] Šrám RJ, Binková BB, Dejmek J, Bobak M (2005). Ambient air pollution and pregnancy outcomes: a review of the literature.. Environ Health Perspect.

[r31] U.S. EPA (U.S. Environmental Protection Agency) (1994). Amendments to 1990 Clean Air Act-List of 189 Hazardous Air Pollutants.

[r32] U.S. EPA (U.S. Environmental Protection Agency) (1999). Determination of Polycyclic Aromatic Hydrocarbons (PAHs) in Ambient Air Using Gas Chromatography/mass Spectrometry (GC/mS). Compendium Method TO-13, EPA//625/R-96/010b.

[r33] U.S. EPA (U.S. Environmental Protection Agency) (2000). Assigning Values to Non-detected/Non-quantified Pesticide Residues in Human Health Food Exposure Assessments.

[r34] WHO (World Health Organization) (1993). Guidelines for Drinking-Water Quality. Chemical Aspects. 2nd ed.. http://www.who.int/water_sanitation_health/dwq/gdwq2v1/en.

[r35] World Health Organization (2000). Air Quality Guidelines for Europe. 2nd ed.

[r36] WHO–IPCS (World Health Organization–International Programme on Chemical Safety) (1998). Selected Non-heterocyclic Polycyclic Aromatic Hydrocarbons. Environmental Health Criteria 202.. http://www.inchem.org/documents/ehc/ehc/ehc202.htm.

