# Supplementary material for: Risk Assessment Related to Atmospheric Polycyclic Aromatic Hydrocarbons in Gas and Particle Phases near Industrial Sites
Source: Environ Health Perspect. 2011 Apr 8;119(8):1110–6. doi: 10.1289/ehp.1002855 (PMC3237345; doi:10.1289/ehp.1002855)
Supplement: (164 KB) PDF [file ehp.1002855.s001.pdf]

**Supplemental Material**

# Risk Assessment Related to Atmospheric Polycyclic Aromatic Hydrocarbons in Gas and Particle Phases near Industrial Sites

Noelia Ramírez[1], Anna Cuadras[2], Enric Rovira[2], Rosa Maria Marcé[1], Francesc Borrull[1]*

[1] Department of Analytical Chemistry and Organic Chemistry, Universitat Rovira i Virgili, Tarragona, Spain

[2] Observatory of Health and Environment of Tarragona, Agència de Protecció de la Salut, Departament de Salut, Generalitat de Catalunya,Tarragona, Spain

* Corresponding author: Professor Francesc Borrull, Department of Analytical Chemistry and Organic Chemistry, Universitat Rovira i Virgili, Marcel·lí Domingo s/n, 43007 Tarragona (Spain). Telephone: + 34977559560. Fax: + 34977558446. E-mail: francesc.borrull@urv.cat

**Supplemental Material, Table 1**. Main method parameters tested by spiking different amounts of the standards in clean PUFs and QFFs and then extracted and analyzed in the optimized conditions. The table shows the average results for extraction of PUFs and QFFs.

| PAHs | Recovery [a] (%, n=5) | Repeatability[b] (%RSD, n=5) | Reproducibility [c] (%RSD, n=5) | LOD[d] (ng $m^{-3}$) | LOQ[e] (ng $m^{-3}$) |
|---|---|---|---|---|---|
| Nap | 90 | 1 | 3 | 0.016 | 0.033 |
| AcPy | 91 | 1 | 1 | 0.033 | 0.100 |
| AcP | 95 | 2 | 2 | 0.016 | 0.033 |
| Flu | 92 | 1 | 2 | 0.033 | 0.167 |
| PA | 98 | 2 | 2 | 0.033 | 0.067 |
| Ant | 97 | 1 | 1 | 0.033 | 0.100 |
| FluT | 91 | 5 | 7 | 0.033 | 0.067 |
| Pyr | 100 | 5 | 6 | 0.002 | 0.017 |
| BaA | 95 | 3 | 4 | 0.002 | 0.017 |
| Chr | 97 | 2 | 2 | 0.002 | 0.017 |
| BbF | 92 | 3 | 5 | 0.016 | 0.033 |
| BjF | 90 | 5 | 10 | 0.002 | 0.017 |
| BkF | 90 | 5 | 10 | 0.002 | 0.017 |
| BeP | 96 | 4 | 7 | 0.002 | 0.017 |
| BaP | 99 | 4 | 8 | 0.033 | 0.167 |
| Ind | 98 | 6 | 9 | 0.033 | 0.167 |
| DahA | 97 | 1 | 5 | 0.033 | 0.167 |
| BghiP | 98 | 6 | 6 | 0.033 | 0.167 |

[a] Recovery calculated as the percentage of the response obtained by the analysis of 5 PUFs and QFFs spiked with 1mL of standard target PAH solution of 1 ppm, compared with the responses obtained by direct injection of the same amount of standard under the same analytical conditions.

[b] Repeatability expressed as relative standard deviation (%RSD) for the analysis of PUFs and QFFs spiked with 1mL of standard target PAH solution of 1 ppm (n=5) on the same day.

[c] Reproducibility expressed as relative standard deviation (%RSD) for the analysis of PUFs and QFFs spiked with 1mL of standard target PAH solution of 1 ppm (n=5) on different days.

[d] Limits of detection (LODs) defined as the concentration equivalent to three times the noise of the quantifier ion, for 300 $m^2$ of sample volume.

[e] Limits of quantification (LOQs) defined as the lower calibration level for each compound, for 300 $m^2$ of sample volume.

**Supplemental Material, Table 2.** Daily PAH values, expressed in ng $m^{-3}$, obtained in Site 1 between the 2nd and the 16th of June 2008.

| PAHs | 2 Jun 08 | | 3 Jun 08 | | 4 Jun 08 | | 10 Jun 08 | | 11 Jun 08 | | 12 Jun 08 | | 16 Jun 08 | |
|---|---|---|---|---|---|---|---|---|---|---|---|---|---|---|
| | **Gas** | **PM** | **Gas** | **PM** | **Gas** | **PM** | **Gas** | **PM** | **Gas** | **PM** | **Gas** | **PM** | **Gas** | **PM** |
| Nap | 3.44 | 3.28 | 2.41 | n.d. | 3.53 | n.d. | 1.49 | n.d. | n.d. | n.d. | 2.10 | n.d. | n.d. | n.d. |
| AcPy | 2.21 | 2.52 | 1.53 | n.d. | 1.80 | n.d. | 0.98 | n.d. | 0.95 | n.d. | 1.32 | n.d. | n.d. | n.d. |
| AcP | 1.88 | 1.53 | 1.04 | n.d. | 1.09 | n.d. | 0.68 | n.d. | n.d. | n.d. | 0.71 | n.d. | n.d. | n.d. |
| Flu | 1.62 | 0.95 | 1.15 | n.d. | 1.35 | n.d. | 0.58 | 0.30 | 0.61 | n.d. | 0.68 | n.d. | 0.72 | n.d. |
| PA | 1.84 | 1.11 | 1.53 | 0.68 | 2.11 | 0.71 | 1.65 | 0.44 | 1.45 | 0.42 | 2.13 | 0.41 | 2.46 | 0.40 |
| Ant | 1.58 | 1.45 | 1.18 | 1.01 | 1.24 | 1.06 | 0.75 | 0.60 | 0.81 | 0.60 | 0.97 | 0.58 | 0.83 | n.d. |
| FluT | 0.81 | 0.88 | 0.63 | 0.39 | 0.70 | 0.41 | 0.51 | 0.24 | 0.61 | 0.24 | 0.88 | 0.21 | 0.94 | 0.22 |
| Pyr | 0.48 | 3.44 | 0.77 | 0.88 | 0.75 | 0.93 | 0.36 | 0.26 | n.d. | 0.29 | 0.58 | 0.28 | 0.70 | 0.26 |
| BaA | 1.11 | 2.67 | 0.91 | 0.88 | 0.99 | 0.93 | 0.62 | 0.47 | n.d. | 0.46 | 0.57 | 0.44 | 0.43 | 0.47 |
| Chr | 1.26 | 3.44 | 0.66 | 1.18 | 0.72 | 1.24 | 0.27 | 0.33 | n.d. | 0.30 | 0.53 | 0.30 | 0.33 | 0.33 |
| BbF | 0.84 | 2.67 | 0.91 | 0.71 | 1.12 | 0.75 | 1.56 | 0.51 | n.d. | 0.42 | 0.41 | 0.48 | 0.38 | 0.47 |
| BjF/BkF | 1.76 | 2.39 | 1.42 | 1.62 | 1.79 | 1.71 | 1.23 | 1.00 | 1.05 | 0.96 | 0.98 | 0.98 | 0.95 | 0.95 |
| BeP | n.d. | n.d. | n.d. | n.d. | n.d. | n.d. | n.d. | n.d. | n.d. | 0.09 | n.d. | 0.12 | n.d. | 0.09 |
| BaP | 1.02 | 1.36 | 0.82 | 0.82 | 1.11 | 0.86 | 0.52 | 0.48 | 0.59 | 0.45 | 0.51 | 0.45 | n.d. | 0.46 |
| Ind | 1.15 | 2.25 | 0.97 | 0.94 | 0.99 | 0.99 | n.d. | 0.49 | 0.62 | 0.51 | 0.52 | 0.59 | 0.45 | 0.58 |
| DahA | 5.34 | 1.86 | 1.07 | 1.10 | 1.16 | 1.16 | n.d. | 0.66 | 0.68 | 0.64 | 0.65 | 0.71 | n.d. | 0.70 |
| BghiP | 2.19 | 1.53 | 0.63 | 0.85 | 0.78 | 0.89 | n.d. | 0.43 | 0.46 | 0.43 | 0.40 | 0.56 | n.d. | 0.48 |
| **Total PAH** | 28.5 | 33.3 | 17.6 | 11.0 | 21.2 | 11.7 | 11.2 | 6.22 | 7.82 | 5.72 | 13.9 | 6.00 | 8.20 | 5.32 |

n.d., values under the limit of detection of each sample

**Supplemental Material, Table 3.** Daily PAH values, expressed in ng $m^{-3}$, obtained in Site 1 between the 1st and the 16th of December 2008.

| PAHs | 1 Dec 08 | | 2 Dec 08 | | 9 Dec 08 | | 10 Dec 08 | | 11 Dec 08 | | 15 Dec 08 | | 16 Dec 08 | |
|---|---|---|---|---|---|---|---|---|---|---|---|---|---|---|
| | Gas | PM | Gas | PM | Gas | PM | Gas | PM | Gas | PM | Gas | PM | Gas | PM |
| Nap | 2.99 | 0.03 | 7.95 | 0.08 | 2.67 | 0.08 | 5.82 | 0.04 | 12.2 | 0.04 | 7.44 | 0.04 | 20.4 | 0.08 |
| AcPy | 1.43 | n.d. | 4.80 | n.d. | 1.55 | n.d. | 3.35 | n.d. | 8.63 | n.d. | 5.53 | n.d. | 9.03 | n.d. |
| AcP | 0.72 | n.d. | 1.81 | 0.04 | 5.77 | 0.04 | 1.33 | 0.03 | 3.70 | 0.03 | 1.72 | 0.04 | 2.52 | n.d. |
| Flu | 6.58 | 0.16 | 11.7 | n.d. | 6.41 | n.d. | 5.18 | 0.16 | 12.5 | n.d. | 14.4 | n.d. | 13.1 | n.d. |
| PA | 31.6 | 0.39 | 65.1 | 0.38 | 39.1 | 0.27 | 17.0 | 0.39 | 46.3 | 0.22 | 91.1 | 0.32 | 55.8 | 0.46 |
| Ant | 3.18 | 0.16 | 5.28 | 0.20 | 3.61 | 0.17 | 1.98 | 0.19 | 4.72 | 0.16 | 9.00 | 0.24 | 5.08 | 0.24 |
| FluT | 5.80 | 0.22 | 12.0 | 0.41 | 6.87 | 0.24 | 3.42 | 0.35 | 9.68 | 0.20 | 20.4 | 0.56 | 11.7 | 0.65 |
| Pyr | 3.96 | 0.55 | 9.11 | 0.55 | 5.41 | 0.47 | 2.44 | 0.60 | 6.44 | 0.41 | 15.5 | 0.76 | 8.07 | 0.86 |
| BaA | 0.78 | 0.34 | 1.09 | 1.02 | 1.17 | 0.48 | 0.64 | 1.85 | 1.38 | 0.26 | 1.09 | 0.73 | 1.17 | 0.58 |
| Chr | 0.65 | 0.55 | 1.25 | 0.95 | 1.03 | 0.38 | 0.07 | 0.11 | 1.53 | 0.26 | 1.26 | 1.88 | 0.99 | 1.22 |
| BbF | n.d. | <LOQ | n.d. | <LOQ | n.d. | <LOQ | n.d. | <LOQ | n.d. | <LOQ | n.d. | <LOQ | n.d. | <LOQ |
| BjF/BkF | 0.50 | 1.1 | 0.66 | 1.36 | 0.12 | 0.74 | n.d. | 0.44 | n.d. | 0.34 | n.d. | 1.93 | n.d. | 1.56 |
| BeP | 0.34 | 0.29 | 0.36 | 0.24 | 0.32 | 0.24 | n.d. | 0.15 | n.d. | 0.15 | n.d. | 0.38 | n.d. | 0.28 |
| BaP | n.d. | 0.73 | n.d. | 0.91 | n.d. | 0.84 | n.d. | 0.50 | n.d. | 0.36 | n.d. | 1.43 | n.d. | 1.53 |
| Ind | n.d. | 0.62 | n.d. | 0.72 | n.d. | 0.55 | n.d. | 0.29 | n.d. | 0.30 | n.d. | 0.89 | n.d. | 0.69 |
| DahA | n.d. | n.d. | n.d. | 0.41 | n.d. | n.d. | n.d. | n.d. | n.d. | n.d. | n.d. | n.d. | n.d. | 0.34 |
| BghiP | n.d. | 0.66 | n.d. | 0.72 | n.d. | 0.65 | n.d. | 0.30 | n.d. | 0.31 | n.d. | 0.90 | n.d. | 0.85 |
| **Total PAH** | 58.5 | 5.54 | 121 | 7.74 | 74.1 | 4.91 | 41.3 | 5.25 | 107 | 2.89 | 167 | 9.73 | 128 | 9.06 |

n.d., values under the limit of detection of each sample
<LOQ, values under the limit of quantification of each sample
